# Effects of biotic and abiotic factors on phenotypic partitioning of wing morphology and development in *Sclerodermus pupariae* (Hymenoptera: Bethylidae)

**DOI:** 10.1038/srep26408

**Published:** 2016-05-19

**Authors:** Xiaoyi Wang, Ke Wei, Zhongqi Yang, David E. Jennings, Jian J. Duan

**Affiliations:** 1 Key Laboratory of Forest Protection, State Forestry Administration, Research Institute of Forest Ecology, Environment and Protection, Chinese Academy of Forestry, 2 Dongxiaofu, Xiangshan Road, Haidian, Beijing 100091, China; 2 Department of Entomology, University of Maryland, 4112 Plant Sciences Building, College Park, MD 20742, USA; 3 United States Department of Agriculture, Agricultural Research Service, Beneficial Insects Introduction Research Unit, Newark, DE 19713, USA

## Abstract

Wing phenotype polymorphism is commonly observed in insects, yet little is known about the influence of environmental cues on the development or expression of the alternative phenotypes. Here, we report how both biotic and abiotic factors affect the wing morph differentiation of a bethylid parasitoid *Sclerodermus pupariae*. The percentage of winged female parasitoid progeny increased exponentially with temperature between 20 °C to 30 °C. Low intensity light and short-day photoperiod conditions also significantly induced the development of winged morphs. Interestingly, wingless maternal parasitoids produced more winged progeny. Furthermore, the degree of wing dimorphism was significantly influenced by the interactions between light intensity and maternal wing morphs. The percentage of winged female progeny was not significantly influenced by foundress densities, but increased significantly with parasitoid brood sizes. However, the percentage of male progeny increased significantly with the densities of maternal parasitoids. Our findings highlight the phenotypic partitioning of wing morphology and development in the parasitoid *S. pupariae* under varied environmental cues, and reveal the most favourable conditions for the production of winged females in this bethylid wasp. It is thus possible to increase winged female parasitoid production for the purposes of biological control by manipulation of biotic and abiotic conditions.

Phenotypic plasticity is a phenomenon in which the same genotype produces entirely different phenotypes in response to environmental cues, granting an organism the ability to adapt to environmental changes^1^,^2^. This evolutionary capacity of a population can affect critical life-history traits, including physiology (*e.g.*, fecundity)^3^, behaviour (*e.g.*, taxis)^4^, and morphology (*e.g.*, body size, wing morph)^5^,^6^,^7^,^8^,^9^. Wing dimorphism is one of the most obvious traits in morph differentiation of an organism associated with phenotypic plasticity. Wing polymorphism is commonly observed in many insect families, as a result of trade-offs between flight capability and fecundity^1^,^10^. For example, when local conditions are relatively stable or predictable and support production of offspring, theory suggests that individuals can obtain higher fitness by investing self-energy in reproduction over dispersal. However, organisms may favour dispersal to new habitats when they encounter deteriorating local environments^11^. Therefore, wing polymorphism can be a highly adaptive trait in insects that live in diverse environmental conditions affecting reproduction.

Currently, there is limited knowledge of the mechanisms responsible for wing polymorphism in insects, even though wing dimorphism has broad implications for theoretical and applied ecology and evolution. Both genetic architecture and environmental factors can affect the developmental outcomes of insect wing morphs^8^,^10^,^11^,^12^,^13^,^14^,^15^,^16^. Indeed, studies in evolutionary and developmental biology have revealed the contributions of genetic architecture to morphological variation. Although the environmental factors influencing wing plasticity also have been identified, how these cues trigger a phenotypic response remains largely unresolved^11^, particularly in Hymenoptera. To date, studies on insect wing polymorphism induced by heterogeneous environments are reported mostly for phytophagous insects, *e.g*., aphids^5^,^8^,^17^,^18^,^19^, crickets^20^,^21^, locusts^22^, planthoppers^23^, and chinch bugs^24^,^25^. A paucity of investigations has focused on parasitic insects; merely, studies on the ectoparasitoid *Melittobia digitata* Dahms indicates that clutch sizes and the nutrient quality are the most important factors influencing its wing dimorphism^7^. The wing dimorphism of this parasitic wasp is considered a trade-off between host resource exploitation and migration^26^. Beyond this, the effects of environmental factors on wing dimorphism in parasitic wasps remain largely unknown.

Sex allocation is the provision of resources to male versus female offspring^27^, and it has been subject to extensive study in parasitoid wasps^27^,^28^,^29^,^30^. This is largely because of the strong and direct links between sex allocation behaviour and fitness consequences. For parasitoid wasps, males develop from unfertilized eggs while females from fertilized eggs under most circumstances. Thus, an ovipositing female can determine the sex of a particular offspring by whether or not she fertilizes the egg in response to local conditions^31^. Among the local conditions influencing sex allocation is local mate competition (LMC)^30^,^32^,^33^,^34^. The theory of LMC indicates that when mating occurs between the offspring of one or multiple mothers, the fraternal competition for mates can lead to an extremely female-biased sex ratio^32^. This female bias can directly reduce the competition for mates among male individuals^33^, although the female-biased sex ratios in parasitoid wasps would become less biased with increasing numbers of unrelated individuals^35^. Understanding sex allocation in parasitoid wasps is important because many parasitoids are used in biocontrol programs against agricultural and forest pests and their sex ratios can affect the success or failure in controlling the target pests^36^. One of the costs of biological control is the additional production of useless males during the mass-rearing of parasitoids. Thus, considerable economic benefits can be gained from manipulating parasitoid wasps’ sex ratio.

*Sclerodermus pupariae* Yang *et* Yao (Hymenoptera: Bethylidae) is a newly discovered native gregarious idiobiont ectoparasitoid of the emerald ash borer (EAB), *Agrilus planipennis* Fairmaire (Coleoptera: Buprestidae) from Tianjin, China^37^. It kills or paralyses mature EAB larvae and pupae (Supplementary Fig. 1) during oviposition on the exterior of the host body^37^. As a generalist, *S. pupariae* also parasitizes cerambycid larvae, including *Anoplophora glabripennis* (Motschulsky), *Apriona swainsoni* (Hope), *Massicus raddei* (Blessig), and *Monochamus alternatus* (Hope) (Coleoptera: Cerambycidae)^37^,^38^,^39^,^40^. This parasitoid is now widely used as a biological control agent against forest wood boring insect pests in China^41^. In biological control programs, although winged and wingless parasitoids can effectively find and parasitize hosts, winged individuals can more easily disperse to colonize habitats via long distance flights beyond the release site. Predictably, releases of winged natural enemies with high dispersal would reduce the overall pest management costs. Female parasitoids of *S. pupariae* can develop into two different morphs (winged or wingless) (Supplementary Fig. 2); however, winged females are only occasionally found in artificially reared populations. Under laboratory conditions, unmated females produce only male offspring, while mated females can produce both male and female progeny. A female-biased sex ratio of this parasitoid has been found in previous studies^9^,^37^, as well as in a congeneric species, *S. harmandi*^35^; however, there are limited data regarding the sex ratios of this species when developing under variable conditions. Moreover, maternal care behaviours such as ensuring eggs and young larvae stay on the host, and remaining with their offspring until pupation/emergence, were recorded in *S. harmandi*^42^. Guarding behaviours similar to those displayed by this congener were also observed in our artificial rearing of *S. pupariae* (K. Wei personal observation).

Numerous cues may influence the expression of insect wing polyphenism, including genetics, population density, host quality, temperature, photoperiod, pheromones and interactions with natural enemies^43^. In this study, we aimed to determine wing morph differentiation of *S. pupariae* progeny and elucidate the mechanisms responsible for wing polymorphism in order to understand the roles of critical environmental cues driving wing polymorphism. Moreover, for the purpose of biological control, a female-biased sex ratio is clearly highly desirable as it is females which are responsible for reducing the pest species’ population size^27^,^44^. Thus, we also need to determine if there are any changes in the sex ratio of the parasitic wasp in association of the development of different wing morphs.

Here, we examine different biotic and abiotic factors that could affect insect wing morph differentiation and development in *S. pupariae*. Parasitoid density has largely been considered to influence the wing development in insects^43^. Consequently, we presumed that high density in the parasitoid juvenile stage would induce more winged individuals to be produced. Furthermore, because the relatively high percentage of winged progeny is typically observed in the first generation after overwintering^38^, we hypothesized that winged females would produce more winged progeny through the potential hereditability of phenotypic traits. Studies on the biology of *S. pupariae* also indicate that adult females prefer dark conditions^38^; hence, long-day photoperiods and high intensity light conditions are considered more apt to induce the development of winged progeny for the dispersal of parasitoids from a deteriorating environment. Indeed, the specific objectives of this study were to (i) examine the effects of both biotic (maternal wing morph variation, parasitoid population density, and brood sizes) and abiotic (temperature, photoperiod, and light intensity) factors on wing morph determination in *S. pupariae*; (ii) assess the interactions among maternal wing morph, photoperiod, and light intensity on female progeny wing morph determination; (iii) determine how sex ratio and developmental duration of parasitoid progeny vary under different conditions; and (iv) analyse the relationship between female parasitoid wing morph and fecundity.

## Results

### Effects of temperature

**Figure 1:**
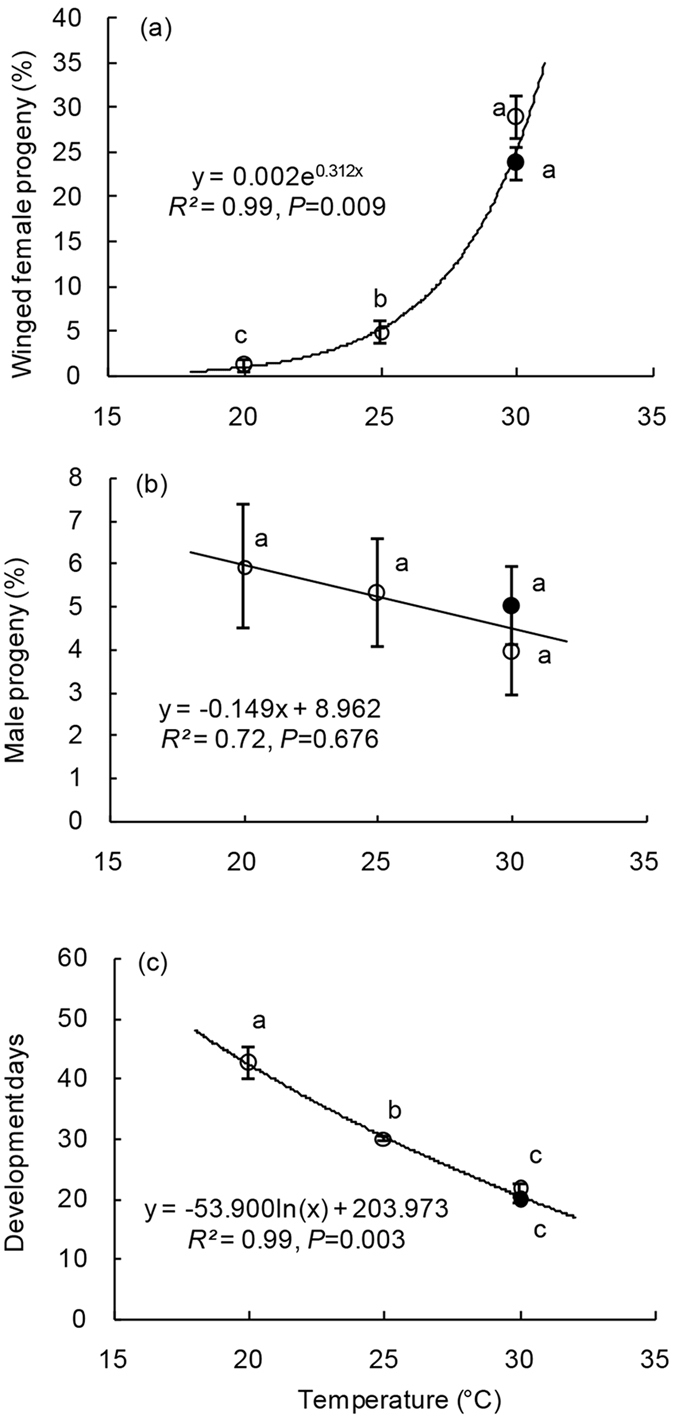
Effects of temperature on progeny wing dimorphism, sex ratio and development duration. (**a**) Percentages of winged female progeny and (**b**) percentages of male progeny with the same letters show no significant differences among treatments according to likelihood ratio *χ*^2^ test at α = 0.05. (**c**) Development days with different letters show significant differences among treatments according to DMRT (Tukey test) at α = 0.05. White and black dots represent samples from the short and the long photoperiod treatments under 30 °C, respectively. Data in the figures are means ± s.e.m.

Female parasitoids did not lay any eggs at 35 °C during long-day and short-day photoperiods, nor at 20 °C in long-day photoperiods; thus, data from these three treatments were excluded in statistical analyses. Proportions of winged female progeny showed a marked exponential relationship with temperature (general linear model, GLM: *df* = 1, 2, *F* = 452.42, *P* = 0.002) (Fig. 1a). Results from logistic regression analyses also indicated that temperature significantly affected the production of wings (nominal logistic regression, NLR: *df* = 2, likelihood χ^2^ = 157.08, *P* < 0.001); the proportion of winged females in the 30 °C treatment was significantly higher than that of treatments at 25 °C and 20 °C (Fig. 1a). These results revealed that temperature had a notable impact on the rate of reproduction of winged progeny in this bethylid wasp, and that high temperatures contribute to wing development in parasitoids. However, the sex ratio of parasitoid progeny showed no significant change among treatment groups (NLR: *df* = 2, likelihood χ^2^ = 0.89, *P* = 0.642) (Fig. 1b).

Temperature significantly impacted the developmental duration of parasitoid progeny (ANOVA: *df* = 2, 37, *F* = 93.85, *P* < 0.001, based on brood), with a pronounced logarithmic relationship between the two variables (GLM: *df* = 1, 2, *F* = 343.47, *P* = 0.003) (Fig. 1c).

### Effects of maternal parasitoid density

**Figure 2:**
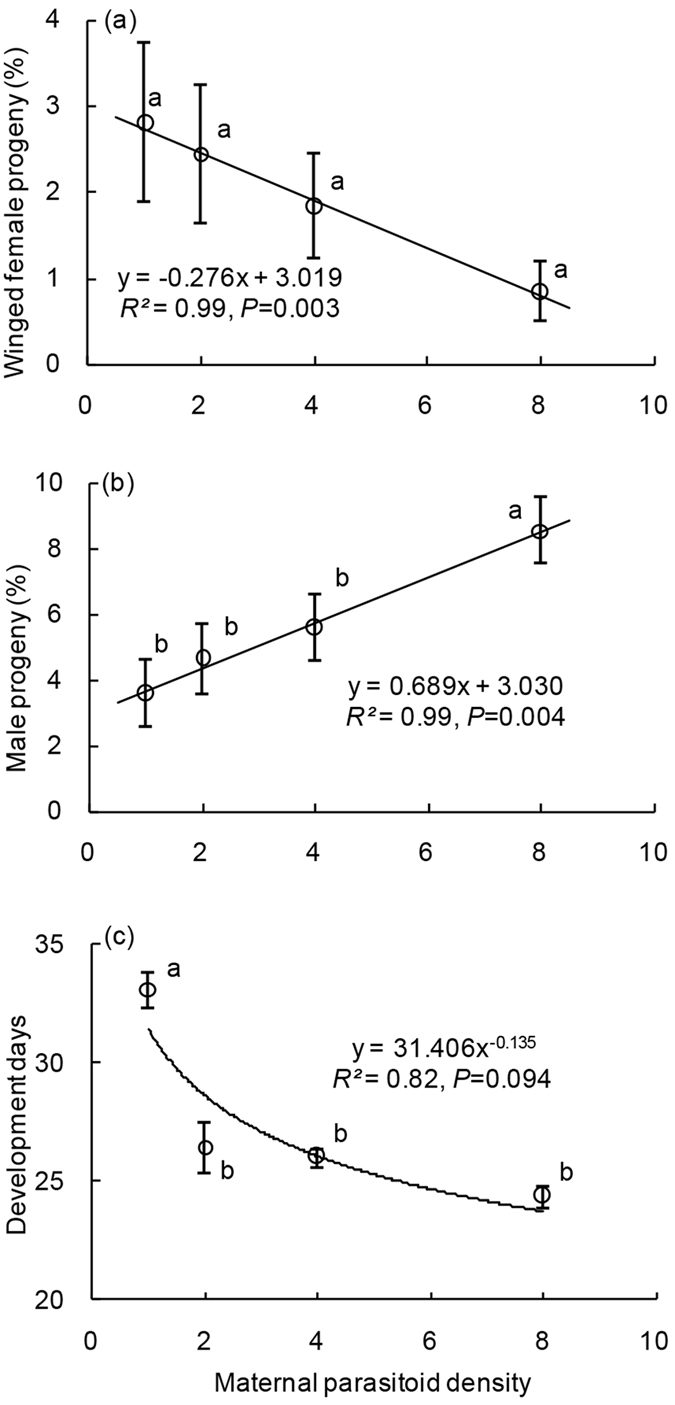
Effects of adult parasitoid density on progeny development. See Fig. 1 legend for interpretation.

Although there was a significant linear relationship between percentages of winged female progeny and maternal densities (GLM: *df* = 1, 2, *F* = 353.59, *P* = 0.003), post hoc testing revealed no significant difference in the proportion of winged females among varying densities of maternal treatments (NLR: *df* = 3, likelihood χ^2^ = 6.76, *P* = 0.080) (Fig. 2a). The progeny sex ratio had a significant linear association with maternal parasitoid density (GLM: *df* = 1, 2, *F* = 265.21, *P* = 0.004) (Fig. 2b), and the percentage of male progeny at high maternal parasitoid density (8 adults per vial) was significantly higher than that at low maternal parasitoid density (1–4 adults per vial) (NLR: *df* = 3, likelihood χ^2^ = 13.26, *P* = 0.004) (Fig. 2b). The progeny developmental durations decreased with maternal parasitoid density (GLM: *df* = 1, 2, *F* = 9.16, *P* = 0.094) (Fig. 2c). Moreover, progeny where one adult parasitoid was included per vial required significantly more time to complete development than did high maternal parasitoid density treatments (ANOVA: *df* = 3, 43, *F* = 29.31, *P* < 0.001, based on brood) (Fig. 2c).

### Effects of light intensity, photoperiod, and maternal wing morph

**Figure 3:**
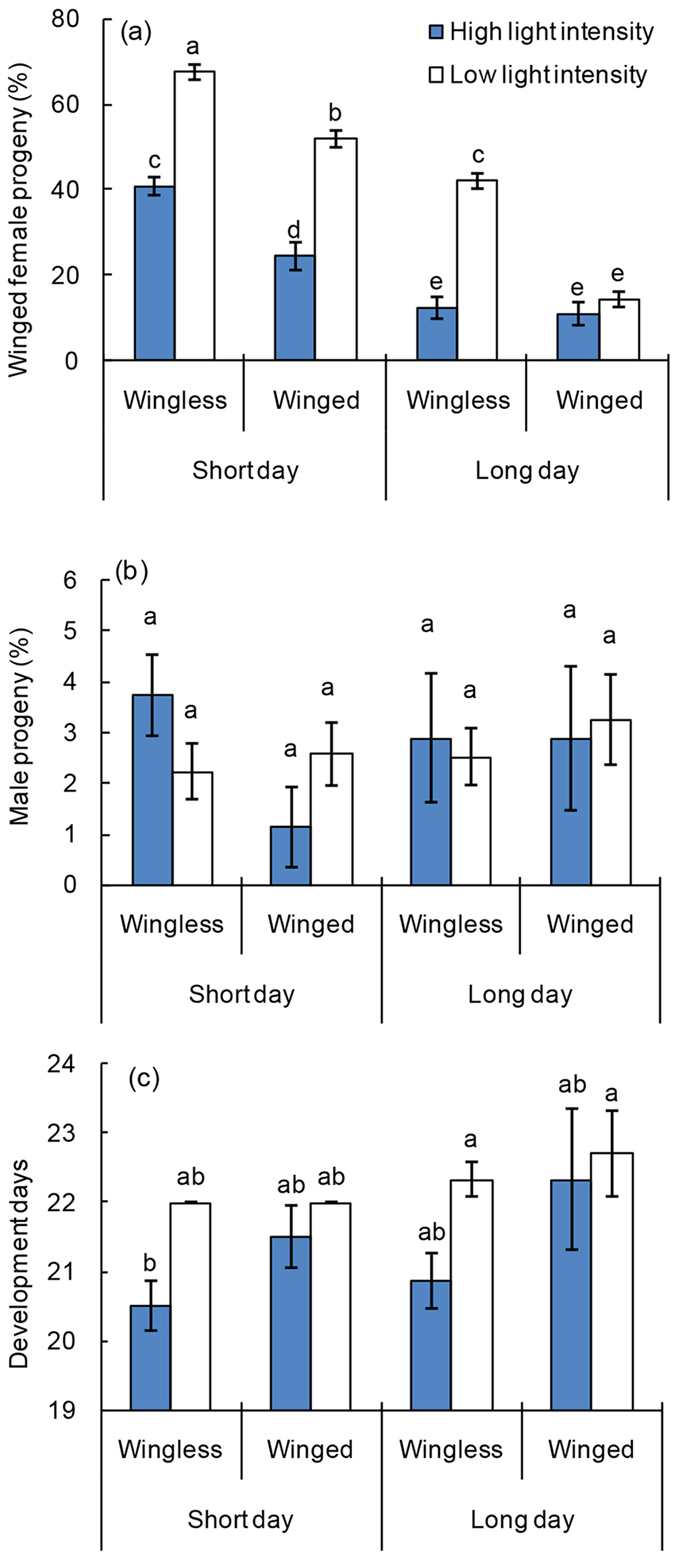
Effects of light intensity, photoperiod and maternal wing morph on progeny development. See Fig. 1 legend for interpretation.

Light intensity, photoperiod, and maternal wing morph had significant effects on the probability of *S. pupariae* female progeny developing into winged adults (NLR: *df* = 7, likelihood χ^2^ = 507.63, *P* < 0.001). Low intensity light was helpful to wing development of progeny (NLR: *df* = 1, likelihood χ^2^ = 102.76, *P* < 0.001), as the wasp progeny more easily developed into winged individuals under a short-day photoperiod, *i.e.*, dark conditions (NLR: *df* = 1, likelihood χ^2^ = 168.25, *P* < 0.001), while wingless maternal parasitoids were inclined to produce winged progeny (NLR: *df* = 1, likelihood χ^2^ = 43.83, *P* < 0.001) (Fig. 3a). Further, light intensity and maternal wing morphs had a significant interaction on parasitoid wing dimorphism (NLR: *df* = 1, likelihood χ^2^ = 7.06, *P* = 0.008), and the effects of light intensity on wingless maternal parasitoids were stronger than winged ones. At the low intensity light condition, the percentages of winged progeny from the same wing morph of maternal parasitoids were significantly higher than that at the high intensity light condition (Fig. 3a). However, the sex ratio of parasitoids that developed from different conditions exhibited no significant difference (NLR: *df* = 7, likelihood χ^2^ = 5.41, *P* = 0.610) (Fig. 3b).

The developmental time of parasitoid progeny differed significantly among treatments (ANOVA: *df* = 7, 79, *F* = 3.97, *P* < 0.001, based on brood) (Fig. 3c). Light intensity also significantly affected the developmental time of progeny (ANOVA: *df* = 1, 85, *F* = 14.23, *P* < 0.001); the development times of parasitoids were delayed approximately one day under low intensity light conditions. Moreover, the parasitoids developed more rapidly under short-day photoperiods than long-day treatments (ANOVA: *df* = 1, 85, *F* = 4.61, *P* = 0.035). However, the development of parasitoid progeny was not significantly influenced by maternal wing morphs (ANOVA: *df* = 1, 85, *F* = 3.49, *P* = 0.065) (Fig. 3c).

### Correlations between brood size and wing dimorphism, sex ratio, and development time

**Figure 4:**
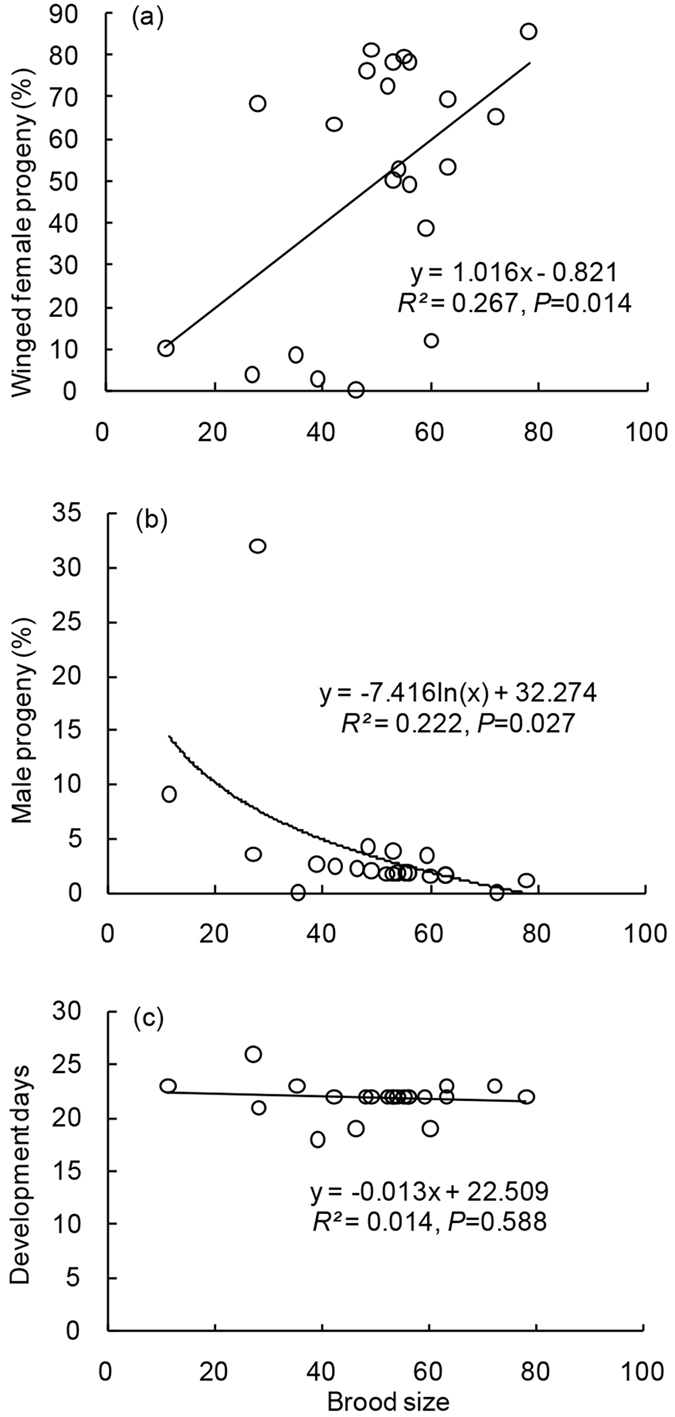
Relationships between (**a**) brood size and progeny wing dimorphism, (**b**) brood size and sex ratio, and (**c**) brood size and development duration of parasitoid *S. pupariae.*

The percentages of winged female progeny increased significantly with parasitoid brood sizes (GLM: *df* = 1, 20, *F* = 7.32, *P* = 0.014) (Fig. 4a), while the sex ratio of progeny showed a negative correlation with brood size (GLM: *df* = 1, 20, *F* = 5.71, *P* = 0.027) (Fig. 4b). Duration of progeny development was not associated with brood size (GLM: *df* = 1, 20, *F* = 0.30, *P* = 0.588) (Fig. 4c).

### Relationship between female parasitoid wing morph and fecundity

**Figure 5:**
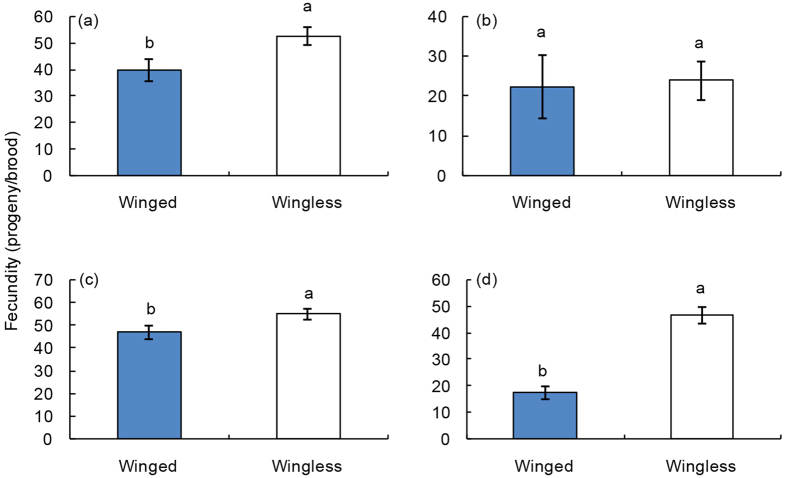
Relationship between maternal wing morph and fecundity of female parasitoids. Shown are (**a**) parasitoids developing from the long photoperiod-low intensity light condition, (**b**) parasitoids developing from the long photoperiod-high intensity light condition, (**c**) parasitoid developing from the short photoperiod-low intensity light condition, and (**d**) parasitoids developing from the short photoperiod-high intensity light condition. The different letters on the top of columns show significant difference in fecundity between winged and wingless maternal parasitoids according to DMRT (Tukey test) at α = 0.05. Data in the figures are means ± s.e.m.

Generally, the fecundity of winged female parasitoids decreased significantly compared with wingless female parasitoids (ANOVA: *df* = 1, 23, *F* = 5.76, *P* = 0.025 for the long photoperiod-low intensity light condition; *df* = 1, 25, *F* = 4.32, *P* = 0.048 for short photoperiod-low intensity light treatment; and *df* = 1, 20, *F* = 51.08, *P* < 0.001 for the short photoperiod-high intensity light condition) (Fig. 5a,c,d). However, the fecundities of these two morphs of female oviposition exhibited no significant difference under the long photoperiod-high intensity light condition (ANOVA: *df* = 1, 11, *F* = 0.03, *P* = 0.871) (Fig. 5b).

### Relationship between sex ratio and wing dimorphism in progeny

**Figure 6:**
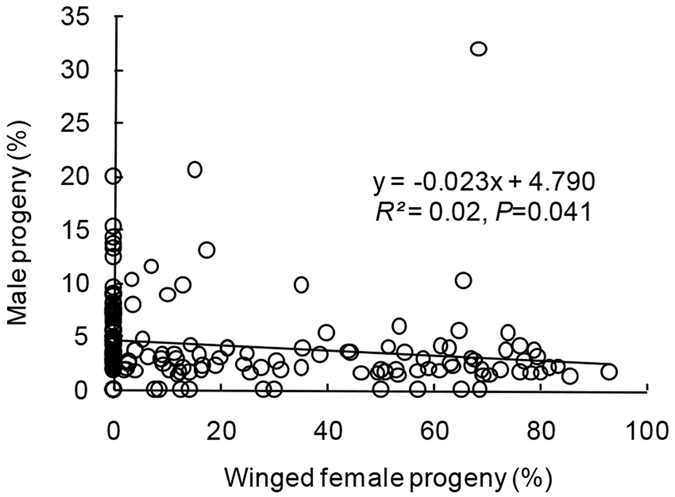


A regression analysis by using overall data from all experiments revealed a significant negative correlation between male percentages and female winged progeny proportions in parasitoid broods (linear regression: *df* = 1,172, *F* = 4.25, *P* = 0.041) (Fig. 6).

## Discussion

In this study, we found that environmental factors of high temperature (30 °C), short photoperiod, low intensity light, maternal wing morph, and intraspecific competition during the larval stage increased production of winged offspring in *S. pupariae* (Table 1). In contrast, foundress density did not affect the wing phenotypes of progeny (Table 1). In general, temperature is a stimulatory factor of insect wing differentiation; however, its effect may be species-specific. High temperatures favour the production of alate or macropter individuals in many species such as *Nilaparvata lugens* (Stål) (Homoptera: Delphacidae)^45^, *Gryllodes supplicans* (Walker) (Orthoptera: Gryllidae)^20^, *Hypera postica* (Gyllenhal) (Coleoptera: Curculionidae)^46^, *Callosobruchus maculatus* (Fabricius) (Coleoptera: Bruchidae)^47^, *Cavelerius saccharivoru*s (Okajima) (Heteroptera: Blissidae)^24^, *Nysius huttoni* White (Heteroptera: Lygaeidae)^48^, and *Orgyia thyellina* (Butler) (Lepidoptera: Lymantriidae)^49^. Despite the widespread evidence in support of high temperatures inducing production of winged individuals, there are exceptions, *e.g*., *Myzus persicae* (Sulzer) and *Lipaphis erysimi* (Kaltenbach) (Homoptera: Aphididae) produced more winged individuals under low temperature conditions^50^. In the present study, the proportion of winged *S. pupariae* females increased with increasing temperature from 20 °C to 30 °C. Parasitoids under the same temperature (30 °C) but varying photoperiods displayed significantly different wing development patterns. These results demonstrated that high temperature, combined with short photoperiod, could stimulate the production of more winged females. Typically, maternal wasps subjected to long photoperiods at 20 °C did not lay eggs, which indicated that photoperiod was an important determinant in the development of this parasitic wasp. Temperature treatments at 35°C were also performed in our experiments, but parasitoids did not lay eggs or immature parasitoids were not able to complete development under these conditions.

The effects of photoperiod and light intensity on wing formation suggest that wing polymorphism evolved as an ability for seasonal adaptation. Short photoperiod and low intensity light were revealed in this study to be advantageous conditions for wing growth in *S. pupariae*. We deduced that the short-day and low intensity light during the overwintering period (late autumn to early spring) experienced by maternal female *S. pupariae* stimulated the production of winged progeny. We also considered that the potential reproduction of parasitoid populations would be slightly reduced in these seasons since we found the wingless parasitoids were more fertile than the winged individuals. Currently, most research regards long photoperiod as a stimulatory cue for wing development, particularly when combined with high temperature^21^,^24^,^51^. The parasitoid *S. pupariae* attacks concealed hosts beneath bark; theoretically, a shorter photoperiod may be more favourable for their development. If we consider the production of dispersal morphs as an evolutionary adaptation to deteriorated environments, more winged females would be produced under long photoperiod and high intensity light conditions. Since our findings do not support our original hypothesis, the mechanisms of light cues on the production of wing phenotypes in this parasitoid require further study.

The proportion of winged individuals increased gradually with larger brood sizes during the larval stage. Currently, intraspecific competition is associated with the production of winged morphs^11^,^52^. Wing polymorphism may in some cases be a graded response to increasing population density. Under a relatively constricted space, a pronounced reduction in food quantity and quality at high consumer density may encourage individuals to migrate. For idiobiont parasitoids, the quantity of resources available for development of progeny is constant at the time of parasitism. Since more larvae develop in one clutch, the nutrient limitation can become more severe, which may trigger production of winged individuals. Interestingly, maternal density did not affect the proportion of winged progeny. An appropriate scenario for this phenomenon is that parasitoids lay reasonable numbers of eggs on hosts by evaluating the hosts’ quality. The egg load of *S. pupariae* depends on host size^9^. Although the maternal parasitoids differ in quantity, the numbers of progeny did not differ significantly among treatments. Therefore, the proportion of winged progeny showed no divergence in this particular experiment.

Developmental time for parasitoid progeny decreased significantly with increasing temperature, maternal parasitoid adult densities, and photoperiodic darkness hours, but not maternal wing morph and brood size. Temperature has an important influence in the development of parasitoids. Developmental times similar to those of *S. pupariae* under different temperatures have been reported for other parasitoids^53^,^54^,^55^. Maternal care may be directly related to the developmental time of this parasitoid. Previous studies of *S. harmandi*, an idiobiont ectoparasitoid, indicated that maternal care is critical to the successful development of its progeny^42^. The females remain on the host after laying eggs until their offspring become adults. Additionally, mothers will move eggs and young larvae back to the hosts if they fall off, and they also remove any microbes colonizing on or near host remains during progeny development. Furthermore, in multiple maternal female experiments, we found the maternal females even take care of progeny produced by other females, not only their own offspring. We presumed that more maternal parasitoids in one vial could take better care of their offspring than that of one female, and development would proceed more rapidly in the situation of more foundresses.

Nutrient limitation may also contribute to the rapid development of this parasitoid^9^. However, developmental time showed no significant difference among the parasitoid brood sizes in our study. Parasitoid oviposition varied on each J-shaped host larva, but this variance may not trigger a different developmental time in this parasitoid. Our findings suggested that nutrient competition in one natural brood can cause wing differentiation but not developmental duration differences in this parasitoid. That these parasitoids develop rapidly under prolonged darkness is consistent with the observation that females prefer ovipositing in darkness. Different development rates may have important consequences for both parasitoid survival and competitive interactions^56^. The slow-growth-high-mortality hypothesis suggests that prolonged development in some insects results in greater exposure to natural enemies and subsequently increases their mortality^57^,^58^. Furthermore, parasitoids that develop faster would have the competitive advantage of being the first to exploit resources^56^. Thus, parasitoids could gain an adaptive advantage through relatively short developmental time frames. The hosts used in our current study were EAB J-shaped mature larvae (late 4^th^ instar), and similar sized hosts were chosen for all experiments. Parasitoid sizes showed no significant difference when using the same grade of sizes of hosts (ZQ Yang, unpublished data). Thus, we assumed that the sizes of parasitoids had no significant differences among treatments in the present study. This principle could be used in the artificial mass production of natural enemies, for instance by accelerating mass production of parasitoids by increasing maternal female density to shorten their generational development duration.

The sex ratio of parasitoid progeny did not differ significantly under biotic or abiotic conditions if there was a single maternal female wasp. We found that exposure of an individual host larva to one female parasitoid always resulted in the emergence of one or a few male offspring. However, when the number of foundresses increased, the percentage of male progeny increased accordingly. Hamilton’s LMC theory indicated that the number of males produced should be maintained at a minimum unless they are required to mate with all available females^32^. When a single wasp parasitizes a host, one male is sufficient to mate with his sisters; therefore, the female only needs to produce one male. However, local resource competition (LRC) also indicated that LMC will be ameliorated or male-biased sex ratios will be favoured in the case of individuals competing for resources (e.g., hosts)^28^. Larval competition within a host is generally asymmetric in gregarious parasitoids^27^. Male individuals are thought to use less host resources to complete development of their immature stages than their female counterparts^59^. Thus, we speculated that more males were produced under high foundress density treatments owing to the judgment of resource limitations from mothers. Under this situation, more sons of a female could also gain some benefits from competition for mating with other female’s daughters. Additionally, we found a negative correlation between sex ratio and the proportion of winged female parasitoids. In fact, this relationship represented the true correlation between sex ratio and parasitoid brood size. As mentioned before, the proportion of winged parasitoids was positively related to parasitoid brood size. This meant that the increment of winged parasitoids, in some cases, represented the enhancement of parasitoid brood size. Hence, the sex ratio showed a declining trend with the increased proportion of winged female parasitoids. Whereas, most of the sex ratios of parasitoids among all experiments were lower than 10% (concentrated in 3–8% with a mean of 4.97%); thus, the correlation between sex ratio and winged female progeny had a lower fit.

The widespread occurrence of flight polymorphisms in insects is consistent with the hypothesis that ‘fitness costs’ are associated with the ability to fly^10^,^60^,^61^,^62^. The energy used to develop wings and flight muscles is simply not available for reproductive investment^10^. Some researchers also noted that delayed development^63^, decreased longevity^64^, and reduced egg size^65^ are often associated with flight capacity in some insects. Additionally, recent work showed that three congeneric hyperparasitoids in the genus *Geilis* (*G. areator, G. agilis, and G. acarorum*) have diverged in wing development and reproduction. *G. areator* develops fully functional wings, whilst *G. agilis* and *G. acarorum* completely lack wings^66^. Reproductive abilities of these three hyperparasitoids varied, with the wingless *G. acarorum* possessing the highest potential fecundity. However, the winged *G. areator* had the highest realised lifetime fecundity^66^. In the present study, wingless bethylid females were generally more fertile than their winged counterparts in the same generation. In some cases, however, we found that when parasitoid females oviposited in long photoperiod-high intensity light conditions, there was no difference among offspring produced from a winged or wingless mother. A potential explanation for this anomalous result could be the relatively small sample size for this treatment. Additionally, we confirmed that parasitoids favour the dark conditions for reproduction^38^. Parasitoids may respond to various cues from unsuitable environmental conditions. Thus, we presumed that the potential difference of fecundity between the two morphs of parasitoids might be weakened by the unsuitable conditions. Moreover, winged or wingless progeny did not vary significantly in time to development. Herein, the relatively low reduction in reproductive abilities of winged individuals would likely be overcome by the need for large quantities of winged females for biological control.

Our findings highlight the phenotypic partitioning of wing morphology and development in the parasitoid *S. pupariae* under varied environmental cues. Moreover, we confirm the conditions favourable for the production of winged female wasps. However, it should be noted that our study maintained constant conditions throughout each experiment, while the daily and seasonal fluctuations in environmental cues in the field would also likely contribute to the outcomes of phenotypic plasticity. In addition, as a few parasitoid progeny may die at immature stages, the results of wing differentiation and sex ratio variance observed in this study are secondary. It is difficult to clarify how many primarily winged or wingless, male or female eggs are produced initially. Another important result of our study was that parasitoids showed rather low oviposition rates in the 1st generation at 20 °C, but increased sharply in the 2nd generation under identical conditions. Additional work is needed to examine if this temperature acclimation is a founder effect or an accidental occurrence among individuals. Furthermore, juvenile hormones have been shown to influence wing polymorphism in insects^10^. Further studies will be focused on the effects of juvenile hormones or precocenes on parasitoid wing morph differentiation.

## Methods

### Parasitoid wasp collection

S*clerodermus pupariae* were collected from mature (J-shaped) EAB larvae (JL) in overwintering chambers on infested *Fraxinus velutina* Torrey (Lamiales: Oleaceae) in Guangang Forest Park, Dagang District, Tianjin City, in April 2011 (38°56′ N, 117°29′ E). The parasitoid colony was established in the laboratory using JL as the host in environmental chambers (Percival Scientific, Perry, IA, USA) under standard conditions of temperature 25 ± 1 °C, 55–65% RH, and photoperiod 16: 8 (L: D). The parasitoid was reared in a small glass vial of 1 cm in diameter and 5 cm in length. Vials were contained in transparent plastic boxes (12 × 7 × 5 cm) covered with aluminium foil to provide low intensity light for parasitoids. Each vial contained a mated female parasitoid adult and a host larva (EAB JL), and vials were sealed tightly with a cotton ball. The parasitoid females were allowed to oviposit on host larvae, and parasitoid progeny developed gregariously in vials. *S. pupariae* adults of the 17th–21st generations were used in all experiments, with a typical age of 1–2 weeks after emergence.

After emergence, parasitoid adults were maintained inside the vials for at least 1 week to provide ample time for mating. Males typically emerged first and mated with females by biting a hole to enter the females’ cocoons. Typically, only one or a few males emerged from a given brood. Neither honey nor water was provided for parasitoid adults prior to experiments. Parasitoid adults fed on haemolymph of host larvae before oviposition. Before experiments, parasitoid adults were maintained in rearing vials inside environmental chambers. The same type of chambers, but with different temperatures and photoperiod conditions, were used for all subsequent experiments.

### Host larvae

EAB larvae were reared from eggs placed on green ash *Fraxinus pennsylvanica* Marshall (Lamiales: Oleaceae) logs (3–10 cm in diameter, 20 cm in length), using methods described in Duan *et al*.^67^. EAB eggs (laid on coffee filter paper) were placed on freshly cut green ash logs against smooth bark surfaces and securing them with Parafilm until hatching. The number of eggs infested on each log varied with log size. Logs infested with EAB eggs were then placed on floral foam bricks (OASIS, Smithers-Oasis Company, Kent, OH, USA) saturated with distilled water and a 0.1% solution of methyl paraben (to prevent mould) within plastic bins (54 × 38 × 29 cm) in environmental chambers at 30 °C. After 4.5–5 weeks, when EAB larvae developed to 3rd/4th instars, logs with host larvae were placed at 25 °C. Three weeks later, these logs were dissected after the EAB developed into mature larvae, *i.e*., JL. The JL were then collected and stored at 2 ± 1 °C for subsequent parasitoid rearing.

### Effects of temperature

To determine the effects of temperature on progeny wing morphs and sex ratios of *S. pupariae*, we used three temperature regimes (20 °C, 30 °C, and 35 °C) in combination with both long-day 16: 8 (L: D) and short-day 8: 16 (L: D) photoperiods. The standard insect rearing conditions (25 °C, photoperiod 16: 8 (L: D), and 55–65% RH) were set as the control (Supplementary Table 1). The temperature ranges were set based on the appropriate conditions during the growing season of the host larvae in field, combined with our laboratory experiences of rearing the parasitoid. *Sclerodermus pupariae* generally attacks cryptic hosts, suggesting that female adults prefer dark conditions for oviposition^38^. Thus, experimental trials for parasitoids were covered by aluminium foil, allowing low intensity light to enter through edges of the bottom cover or crevices. All tests were conducted using the same size glass vials 1 cm in diameter and 5 cm in length. Each vial contained one wingless female parasitoid and one EAB JL. Oviposition and developmental progress of the parasitoid progeny were observed daily. The dates of oviposition, egg hatching, cocooning, and emergence were recorded in replicate. Maternal parasitoids were removed after the progeny cocooned, and cocoons remained under the same conditions for the development of parasitoid progeny. After progeny emergence was completed, the total number of progeny, and numbers of winged and wingless females and males were counted by replicate for each treatment. Each treatment consisted of 15 replicates.

### Effects of maternal parasitoid adult densities

To examine the effects of maternal parasitoid adult densities on wing morphs, sex ratios, and development of progeny, 4 parasitoid densities (treatments) were established (Table S1), *i.e.*, 1, 2, 4, and 8 mated wingless females were introduced to exposure vials, and each vial had one host. Because the maternal female parasitoids used in this experiment emerged at standard rearing conditions (25 °C, 16: 8 (L: D), low intensity light, and 55–65% RH), we conducted this experiment in an environmental chamber under identical conditions to eliminate confounding influences of other factors besides maternal female density. Maternal parasitoids were removed after the progeny cocooned, and the cocoons remained under the same conditions for parasitoid progeny development. After progeny emergence was completed, total numbers of winged and wingless female and male progeny were counted by replicate for each treatment. Each treatment consisted of 15 replicates.

### Effects of light intensity, photoperiod, and maternal wing morph

The effects of light intensity, photoperiod, and maternal wing morphs on wing morph differentiation, sex ratios, and development of progeny were tested under 30 °C and 55–65% RH condition. A total of 8 treatments (2 levels of photoperiod (long- or short-day) × 2 levels of light intensity (low or high) × winged or wingless foundress) were established (Table S1). The same size glass vials (1 cm in diameter and 5 cm in length) as in the previous experiments were used in this experiment. Each vial contained one female parasitoid and one host larva. Oviposition and developmental progress of parasitoid progeny were observed daily. The dates of oviposition, egg hatching, cocooning, and emergence were recorded in replicate. Maternal parasitoids were removed after the progeny cocooned, and cocoons remained under the same conditions for parasitoid progeny development. After progeny emergence was completed, total numbers of winged and wingless female and male progeny were counted by replicate for each treatment. Each treatment consisted of 15 replicates.

### Correlations between brood size, wing dimorphism and sex ratio

To explore the correlations between parasitoid density during the larval stage on progeny wing differentiation and sex ratio patterns, the relationships among brood size and progeny developmental duration, winged female proportions, as well as male proportions were tested. The number of progeny having emerged from a host in each rearing vial was regarded as the brood size for each replicate. In order to keep all test conditions consistent with the exception of brood size, data were collected from 30 °C and short-day photoperiod treatments in the temperature experiment, and short-day-low intensity light-wingless foundress treatments in the light intensity, photoperiod, and maternal wing morph experiment.

### Fecundity of winged and wingless females

To evaluate putative variance in reproductive costs between winged and wingless parasitoids, the relationship between maternal wing morphs and fecundity was analysed. The number of progeny produced from a winged or wingless female in the light intensity, photoperiod, and maternal wing morph experiment were regarded as the fecundity of a female individual. Parasitoids developed from different conditions were separated for analysis.

### Relationship between sex ratio and wing dimorphism in progeny

To explore the interaction of sex ratio and wing dimorphism in broods, a correlation analysis was conducted by using overall data from all experiments. Male proportions and female winged proportions of parasitoid progeny in every brood were calculated by replicates in each experiment.

### Statistical analyses

A nominal logistic regression model was used to analyse the effects of abiotic variables of temperature, light intensity, and photoperiod, as well as biotic variables of maternal wing morphs, maternal parasitoid densities on wing morph and sex ratio differentiation in parasitoid progeny. Likelihood ratio chi-square tests were used to compare winged female progeny percentages in different treatments for each experiment, as well as the sex ratios of parasitoid progeny. Analyses of variance (ANOVA) using PROC GLM, followed by Tukey’s separation of means test, were used to compare the mean developmental days of parasitoid progeny in different treatments for each experiment. ANOVA were also used to compare the difference of fecundity between winged and wingless maternal female parasitoids. Linear (PROC REG) or nonlinear regression (PROC GLM) analyses were used to evaluate the correlations between parasitoid progeny development and biotic/abiotic factors, with winged female progeny percentages, male progeny percentages, progeny development duration as the dependent variables and temperature, maternal parasitoid density, and brood sizes as independent variables. Linear regression (PROC REG) also used to fit the relationship between sex ratio and wing morph differentiation in parasitoid progeny, with male percentage as the dependent variable and winged female proportion as the independent variable. All statistical analyses were performed with SAS software version 10.0.0 (SAS Institute Inc. 2012, Cary, NC, USA).

## Additional Information

**How to cite this article**: Wang, X. *et al*. Effects of biotic and abiotic factors on phenotypic partitioning of wing morphology and development in *Sclerodermus pupariae* (Hymenoptera: Bethylidae). *Sci. Rep.* **6**, 26408; doi: 10.1038/srep26408 (2016).

## Supplementary Material

Supplementary Information

## Figures and Tables

**Table 1:** Summary of the effects of biotic and abiotic factors on the development of parasitoid wing morphs.

**Category**	**Factor**	**Effect on production of winged female progeny**		
		**Positive**	**Negative**	**Significance level**
Biotic	Maternal wing morph	Wingless	Winged	*P* < 0.001 (***)
	Brood size	High	Low	*P* = 0.014 (**)
	Foundress densities	Low	High	*P* = 0.080 (N.S.)
Abiotic	Temperature	High	Low	*P* < 0.001 (***)
	Photoperiod	Short	Long	*P* < 0.001 (***)
	Light intensity	Low	High	*P* < 0.001 (***)

**Means significant at *P* < 0.05, ***means significant at *P* < 0.01, N.S. Means not significant.
