# Supplementary material for: Effects of biotic and abiotic factors on phenotypic partitioning of wing morphology and development in Sclerodermus pupariae (Hymenoptera: Bethylidae)
Source: Sci Rep. 2016 May 19;6:26408. doi: 10.1038/srep26408 (PMC4872219; doi:10.1038/srep26408)
Supplement: Supplementary Information [file srep26408-s1.pdf]

Supplementary Information

Manuscript Title: Effects of biotic and abiotic factors on phenotypic partitioning of wing morphology and development in *Sclerodermus pupariae* (Hymenoptera: Bethylidae)

Author lists: Xiaoyi Wang, Ke Wei, Zhongqi Yang, David E. Jennings and Jian J. Duan

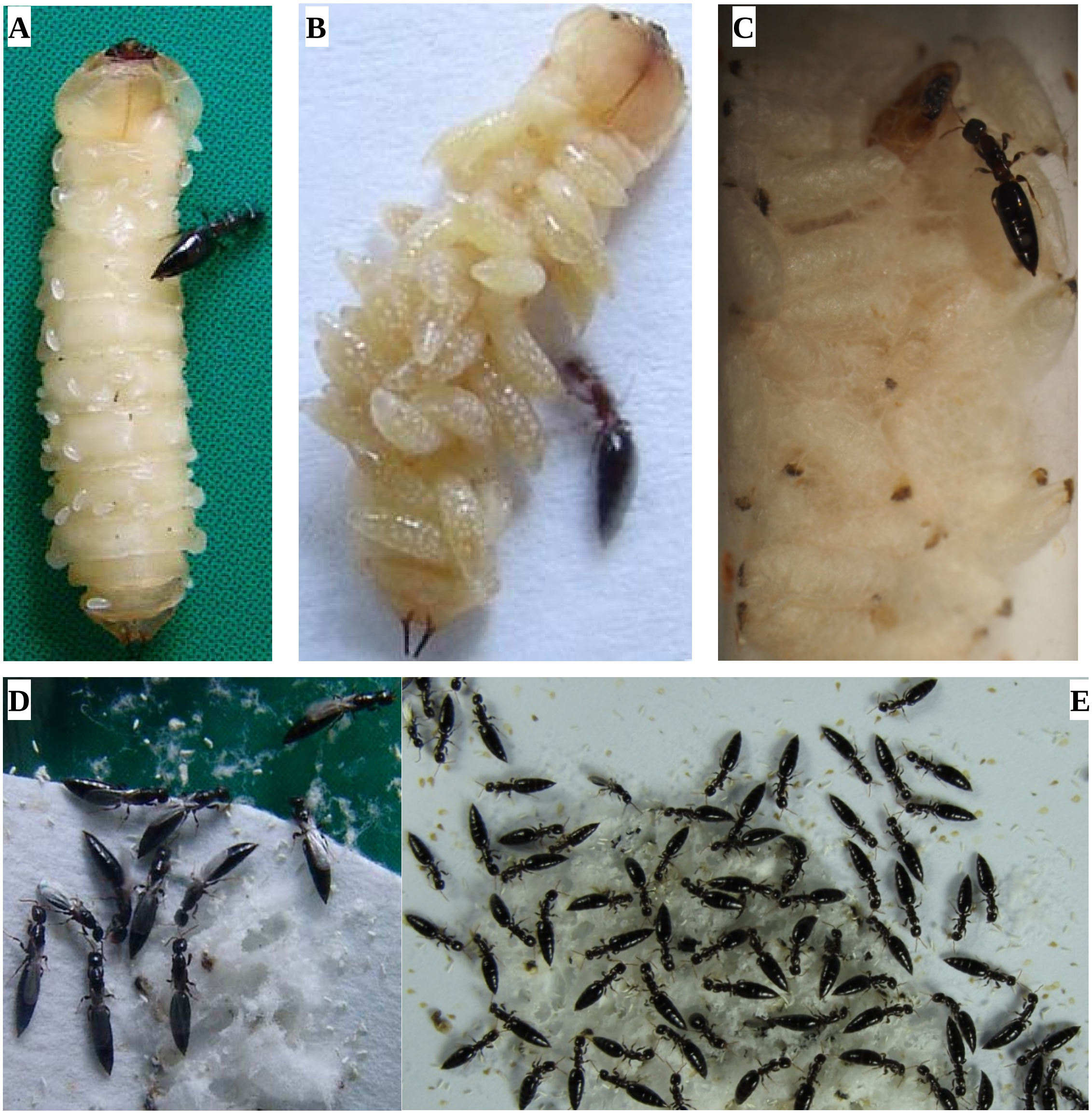

Supplementary Figure 1 *Sclerodermus pupariae* Yang *et* Yao parasitizing mature larvae of emerald ash borer *Agrilus planipennis* Fairmaire. (A) Oviposition; (B) Development of parasitoid larvae; (C) Cocoon; (D) Winged females; (E) Wingless females.

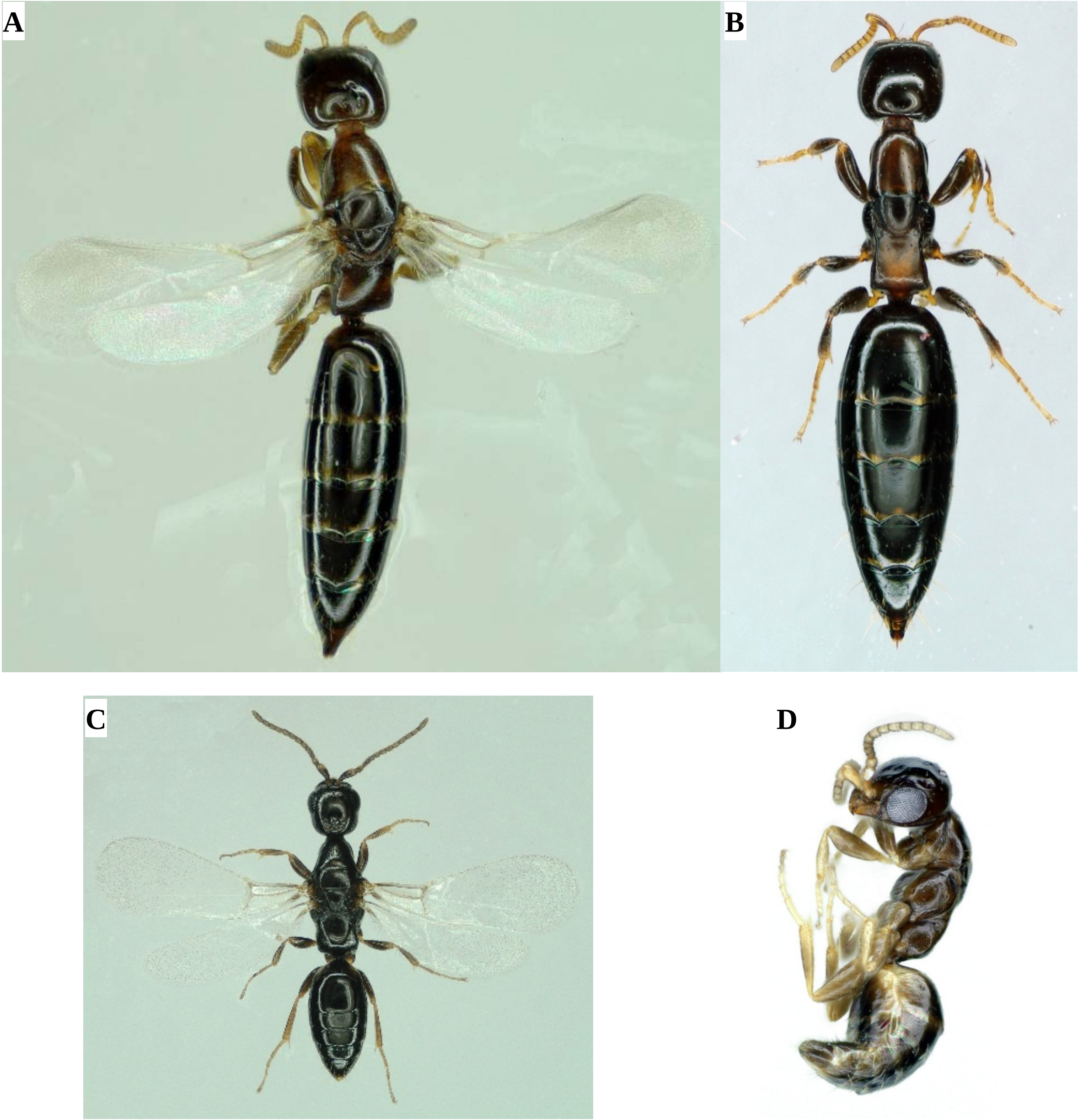

Supplementary Figure 2 Wing morphs of *Sclerodermus pupariae* Yang *et* Yao (Hymenoptera: Bethylidae). (A) Winged female; (B) Wingless female; (C) Winged male; (D) Wingless male.

Supplementary Table 1

Table S1. Experimental designs and examination conditions

Experiment	Temperature (°C)	Photoperiod (L : D) h	Maternal wing morph	Light intensity	Maternal parasitoid adult density	Maternal parasitoid generation
1: Temperature	20	16:8	Wingless	Weak	1	F17
	20	8:16	Wingless	Weak	1	F17-F19
	25	16:8	Wingless	Weak	1	F17
	30	16:8	Wingless	Weak	1	F17
	30	8:16	Wingless	Weak	1	F17
	35	16:8	Wingless	Weak	1	F17
	35	8:16	Wingless	Weak	1	F17
2: Maternal parasitoid adult densities	25	16:8	Wingless	Weak	1	F18
	25	16:8	Wingless	Weak	2	F18
	25	16:8	Wingless	Weak	4	F18
	25	16:8	Wingless	Weak	8	F18
3: Light intensity, photoperiod and maternal wing morph	30	16:8	Winged	Weak	1	F18
	30	16:8	Wingless	Weak	1	F18
	30	8:16	Winged	Weak	1	F18
	30	8:16	Wingless	Weak	1	F18
	30	16:8	Winged	Strong	1	F18
	30	16:8	Wingless	Strong	1	F18
	30	8:16	Winged	Strong	1	F18
	30	8:16	Wingless	Strong	1	F20

Supplementary Table 2

Table S2. Survival from egg to adult of parasitoid throughout experiments

Experiment	Temperature (°C)	Photoperiod (L : D) h	Maternal wing morph	Light intensity	Maternal parasitoid adult density	Number of egg laid	Number of progeny	Survival rate (from egg to adult) (%)	Significance
1. Temperature	20	8:16	Wingless	Weak	1	283	269	95.05	χ^2=0.297, df=3, P=0.961
	25	16:8	Wingless	Weak	1	335	319	95.22	
	30	16:8	Wingless	Weak	1	604	576	95.36	
	30	8:16	Wingless	Weak	1	399	381	95.49	
2: Maternal parasitoid adult densities	25	16:8	Wingless	Weak	1	347	332	95.68	χ^2=0.302, df=3, P=0.960
	25	16:8	Wingless	Weak	2	403	387	96.03	
	25	16:8	Wingless	Weak	4	542	517	95.39	
	25	16:8	Wingless	Weak	8	806	769	95.41	
3: Light intensity, photoperiod and maternal wing morph	30	16:8	Winged	Weak	1	420	400	95.24	χ^2=2.454, df=7, P=0.931
	30	16:8	Wingless	Weak	1	829	793	95.66	
	30	8:16	Winged	Weak	1	686	661	96.36	
	30	8:16	Wingless	Weak	1	754	718	95.23	
	30	16:8	Winged	Strong	1	146	139	95.21	
	30	16:8	Wingless	Strong	1	182	173	95.05	
	30	8:16	Winged	Strong	1	186	177	95.16	
	30	8:16	Wingless	Strong	1	584	563	96.40	
